# Effects of Topical Fucosyl-Lactose, a Milk Oligosaccharide, on Dry Eye Model: An Example of Nutraceutical Candidate

**DOI:** 10.3389/fphar.2015.00280

**Published:** 2015-11-18

**Authors:** Claudio Bucolo, Maria Musumeci, Salvatore Salomone, Giovanni Luca Romano, Gian Marco Leggio, Caterina Gagliano, Michele Reibaldi, Teresio Avitabile, Maurizio G. Uva, Salvatore Musumeci, Filippo Drago

**Affiliations:** ^1^Department of Biomedical and Biotechnological Sciences, Section of Pharmacology, School of Medicine, University of Catania, Catania, Italy; ^2^Department of Laboratory Medicine and Microbiology, Center for Integrated Research, Campus Bio-Medico University of Rome, Rome, Italy; ^3^Department of Chemical Sciences, University of Catania, Catania, Italy; ^4^Institute of Biomolecular Chemistry, The Consiglio Nazionale delle Ricerche, Catania, Italy; ^5^Department of Ophthalmology, School of Medicine, University of Catania, Catania, Italy

**Keywords:** fucosyl-lactose, dry eye, milk oligosaccharides, colostrum, nutraceutical compound

## Abstract

**Purpose:** Colostrum has been proposed to treat severe dryness and problematic eye lesions showing a beneficial effect. The aim of the study was to investigate the effect of 2-fucosyl-lactose, a natural sugar present in the human colostrum, in an experimental dry eye.

**Methods:** Dry eye was induced in adult male New Zealand albino rabbits by topical administration of 1% atropine. Tear volume (Schirmer’s test), tear film breakup time (TBUT), corneal staining and tear osmolarity were assessed. Fucosyl-lactose eye drops was instilled at different concentrations (0.01, 0.1, and, 1%).

**Results:** After 24 h from first atropine administration, tear volume and TBUT values were significantly improved in groups treated with 2-fucosyl-lactose in a dose-dependent manner. Tear volume increased from 5.25 to 10.75 mm and TBUT values from 8.75 to 34.5 s with 0.01% or 1% 2-fucosyl-lactose treatment, respectively. No changes were observed in terms of corneal staining among the all groups treated with 2-fucosyl-lactose. Atropine instillation caused an increase of tear osmolarity (428 mOsm/L), which was reversed by topical treatment with 2-fucosyl-lactose at all doses.

**Conclusion:** The present study demonstrated that 2-fucosyl-lactose, a human milk oligosaccharide, has protective effect on tear film stability.

## Introduction

Dry eye disease, also known as keratoconjunctivitis sicca, is a multifactorial disorder of the tears and ocular surface. Common symptoms of dry eye disease include dryness, irritation, foreign body sensation, light sensitivity, and itching. It is estimated that almost 5 million Americans 50 years and older have dry eye disease, and millions more experience episodic symptoms of dry eye; of these, approximately two-thirds are women (Smith et al., 2007). The prevalence of dry eye disease rises dramatically with increasing age, and as older populations grow, so too will the burden of dry eye disease-associated morbidity. The pathogenesis of dry eye disease is not fully understood; however, it is recognized that hyperosmolarity could affect the surface tension and the stability of the tear film. Hyperosmolarity of tears is an important feature that has been investigated and is one of the strongest predictors of dry eye syndrome (Lemp et al., 2007). Hyperosmolarity environment could contribute to trigger an inflammatory process; it is noteworthy that inflammation has a prominent role in the development and amplification of the signs and symptoms of dry eye disease.

Oligosaccharides are important constituents of the human milk most of all are fucosylated. Among all fucosylated milk oligosaccharides, the α1,2-linked fucosylated glycans, which require the secretor gene for expression in human milk, are the dominant glycan structure found in the milk of secretor mothers (Newburg, 1996; Newburg et al., 2004). Despite the role of oligosaccharides were initially unclear, it has lately become evident that 1,2-fucosylated milk oligosaccharides represent a component of an innate immune system by which the lactating mother protects her infant baby from environmental pathogens, particularly during the first months. Viruses, bacteria and toxins may become pathogenic after adhering to binding sites located on the surface of intestinal epithelial cells (Coyne et al., 2005). Thus, intestinal cells are covered with 1,2 fucosylated glycoproteins and glycolipids protecting by pathogens (Coyne et al., 2005). A mannosyl-fucosyl receptor on the surface of macrophages has been described by Sarkar et al. (1996). The 2-fucosyl-lactose is the most abundant oligosaccharide in the milk (roughly 5 g/L) representing up to 30% of the total human milk oligosaccharides (Erney et al., 2000; Chaturvedi et al., 2001). Interestingly, 2-fucosyl-lactose, the first appearing oligosaccharides in human colostrum, is synchronized with the concomitant macrophage deactivation (Musumeci et al., 2006).

Based on the above evidences, we focused our attention to 2-fucosyl-lactose, a constituent of colostrum, assessing the effect of this sugar on tear film stability in an experimental dry eye model.

## Materials and Methods

### Animals

Male New Zealand albino rabbits weighing 1.8–2.0 kg (Harlan, Italy) were used. Animals were housed in single cage upon arrival in the facilities (in a light and temperature controlled room) with tap water and standard chow provided *ad libitum*. Animal procedures followed guidelines of the Animal Care and Use Committee of the University of Catania, and conformed to the Association for Research in Vision and Ophthalmology (ARVO) resolution on the use of animals in research.

### Dry Eye Model

Atropine sulfate 1.0% ophthalmic solution (Atropina Lux, Allergan, Italy) was instilled into the lower conjunctival sac of each eye three times a day for the duration of each study. We used atropine, a cholinergic (M_3_) receptor antagonist, in order to decrease aqueous production and modify tear stability. Fifteen minutes after atropine instillation we treated the eyes with fucosyl-lactose eye drops or PBS. To assess the degree of dry eye, tear volume, tear film breakup time (TBUT), and corneal staining were used as end points. Baseline values for tear volume, slit lamp examination of tear film, and corneal integrity were obtained 1–2 days before the induction of dry eye according to an established methodology (Shafiee et al., 2011). Tear volume was evaluated by the Schirmer test (Schirmer strips by Eagle Vision, Memphis, TN, USA). The strips were carefully placed in the posterior (i.e., temporal) lower fornix for 60 s, and the wetted area was read in millimeters as an index of tear volume. TBUT was determined after instillation of 5 μl of 2% sodium fluorescein in sterile PBS onto the lower eyelid trying to evenly spread the eye drops on the cornea by manual blinking of the lids. Under slit lamp (Sbisà, Firenze, Italy) with cobalt blue filter to enhance the fluorescein patterns, the time from opening of the eyes until the appearance of the first black spot or streak on the cornea was recorded. The procedure was sequentially performed three times and the average of three readings was generated. Corneal epithelial-staining was evaluated using the National Eye Institute grading system (Lemp, 1995). The corneal surface was divided into five areas, 0–3 staining severity for each area was designated, and the grades were added, providing a total severity of 0–15. All measurements were performed in awake animals in the following sequence: TBUT, corneal staining, and Schirmer test. Tear osmolarity (mOsm/L) was measured, in a separate set of animals, using an Osmomat 30 freezing-point osmometer (Gonotech, Berlin, Germany); tear sample, collected by a glass capillary tube, was diluted and the value generated was then normalized (Aragona et al., 2002). Tear collection was performed in a separate set of animals to avoid any artifact in the assessment of TBUT, corneal staining and Schirmer test. Five groups of animals (four animals/group) were used and treated as following: (1) control (none treatment); (2) vehicle (atropine treatment + eye drops vehicle); (3) 0.01% Fucosyl-lactose (atropine treatment + fucosyl lactose treatment); (4) 0.1% Fucosyl-lactose (atropine treatment + fucosyl lactose treatment); (5) 1% Fucosyl-lactose (atropine treatment + fucosyl lactose treatment).

### Statistical Analysis

All data are expressed as mean ± SD unless indicated otherwise. Statistical analysis was conducted using ANOVA followed by the Dunnett’s test. A *p*-value < 0.05 was considered significant.

## Results

Atropine’s action is related to a pharmacological antagonism to muscarinic receptors in the lacrimal gland that cause a significant decrease of aqueous production with an evident modification of tear stability. It has been described (Stern et al., 1998) that ocular surface (cornea, conjunctiva, and accessory lacrimal glands), Meibomian glands, and main lacrimal gland, are interconnected by neural reflex loops that maintain an integrated “functional unit.” The neural reflex loops involved in maintaining the normal tear physiology can be blocked by anticholinergic agents such as atropine, as demonstrated by Schoenwald et al. (1998) in a rabbit dry eye model.

Multiple topical administration of atropine significantly reduced tear volume (3.75 ± 0.5 mm) compared with baseline (12.5 ± 0.5 mm) after 24 h (Table 1). Fucosyl-lactose (0.01, 0.1, and 1.0%) significantly inhibited atropine-induced tear volume reductions after 24 h in a dose-dependent manner (Table 1). The highest dose of fucosyl-lactose eye drops maintained tear volume near the baseline levels. These data indicate that the treatments completely prevented atropine-mediated effects in tear volume. When evaluating the tear integrity of atropine-treated eyes as measured by TBUT, trends similar to those observed in tear volume were identified.

**TABLE 1:** **Tear volume (Schirmer test) and tear break up time (TBUT) after 24 h from the first atropine administration**.

**No atropine**	**Atropine**			
**Control**	**Vehicle**	**0.01% Fucosyl-lactose**	**0.1% Fucosyl-lactose**	**1% Fucosyl-lactose**
**Schirmer test (mm)**				
12.5 ± 0.5	3.75 ± 0.5*	5.25 ± 0.5*	7.75 ± 0.9*	10.75 ± 0.9*
**TBUT (sec)**				
>60	4.75 ± 0.9*	8.75 ± 0.9*	18.5 ± 1.0*	34.5 ± 1.3*

*p<0.05 vs. vehicle; n=6–8.

Atropine-treated eyes showed a robust reduction in terms of TBUT at 24 h (around 5 s) when compared with control (35 s). Fucosyl-lactose (0.01, 0.1, and 1.0%) significantly inhibited atropine-induced TBUT reduction after 24 h in a dose-dependent manner (Table 1). Fucosyl-lactose (1%) eye drops maintained TBUT at high levels when compared to vehicle group (Table 1). All together these data demonstrated that topical treatment with fucosyl-lactose prevented the effects of atropine on tear stability.

It is also well known that an increased tear film osmolarity causing ocular surface damage, therefore, tear osmolarity was assessed in rabbit with dry eye with or without fucosyl-lactose treatment. Atropine instillation caused a significant increase of tear osmolarity, which was significantly reversed by fucosyllactose topical treatment with a trend that suggest a dose-dependent manner, although there is no significant difference in the three formulations (Table 2).

**TABLE 2:** **Tear osmolarity after 24 h from the first atropine administration**.

**No atropine**	**Atropine**			
**Control**	**Vehicle**	**0.01% Fucosyl-lactose**	**0.1% Fucosyl-lactose**	**1% Fucosyl-lactose**
**Tear osmolarity (mOsm/L)**				
335 ± 12	428 ± 36*	349 ± 29*	330 ± 18*	322 ± 14*

*p<0.05 vs. vehicle; n=6–8.

In the present study corneal staining was not reduced by fucosyllactose in atropine-treated eyes (data not shown).

## Discussion

The results of the present study suggest that 2-fucosyl-lactose eye drops might be effective for the dry eye disease. Fucosyl-lactose improved ocular surface integrity, increased tear volume and restored the tear osmolarity. The ocular surface system consists of the cornea, conjunctiva, lacrimal glands, meibomian glands, nasolacrimal duct, and their associated tear and connective tissue matrices, as well as the eyelids and eyelashes, all integrated by continuous epithelia and interconnected nervous, endocrine, immune, and vascular systems. Factors that perturb the fine homeostatic balance of the ocular surface system can adversely affect tear film stability resulting in inflammatory damage. Tear film instability and tear hyperosmolarity are considered core mechanisms in the development of dry eye. It has been demonstrated that hyperosmolar levels in the tear film may transiently spike during tear instability, resulting in corneal inflammation (Liu et al., 2009). Hyperosmolarity is recognized to be an important mechanism within the etiology of dry eye disease. Hence, hyperosmolarity represents one of the key mechanism to trigger the machinery of ocular surface pathology produced within dry eye syndrome, principally through eliciting inflammation, cell death, and destabilizing the tear film (Lemp et al., 2007). Animal models of dry eye that employ either anti-cholinergic agents and environmental stress show that ocular surface discomfort can induce the inflammatory/T-cell alterations seen in human dry eye. Evidence suggests that inflammation induced by desiccating stress is mediated by T-cells (Knop et al., 2008; Zheng et al., 2010; Zhang et al., 2011). Like other tissues, the eye contains a unique immunoregulatory network designed to limit bystander tissue damage during microbial insults and maintain tolerance to self-antigens and commensal microbes. The eye, like the gut, contains its own local lymphoid tissues, i.e., conjunctiva-associated lymphoid tissue (CALT), situated to sample antigens and maintain tolerance to commensal flora (Newburg et al., 2005; Knop et al., 2008). Alterations in one or several components of the ocular surface system or its secretions results in changes in the tear film or corneal epithelial surface composition (e.g., tear osmolarity and tear volume), leading to susceptibility to desiccation and epithelial damage. In humans tear film osmolarity is increased in all forms of dry eye and tear volume is decreased in aqueous-deficient dry eye. There has been an adequate increase in knowledge regarding the pathophysiology of dry eye in the last years, however there are several unmet points in terms of dry eye treatment. We demonstrated, for the first time, that 2-fucosyl-lactose, a natural sugar present in the human colostrum, prevents tear film instability. Okano and collaborators have recently demonstrated that fucose-containing carbohydrates are able to induce a Th2 response (Okano et al., 2001). It is noteworthy that with different ethnicity there is also different milk oligosaccharide profiles, in particular 2-fucosyl-lactose varies among individuals and over the course of lactation, and may occur in even higher concentrations in colostrum (Coppa et al., 1999). Recently, it has been demonstrated that colostrum from Burkinabe women have significantly higher levels of 2-fucosyl-lactose compared with Italian women colostrum (Sotgiu et al., 2006). A comparable percentage of the secretor genotype for the Lewis blood group phenotype in both Burkinabe and Italian women was found. It is noteworthy that Burkinabe women used to instill some drops of colostrum to the newborn; this represents a community heritage. It has been recently demonstrated that 2-fucosyllactose possess immune-modulating effects, which are able to reduce the proliferation and detrimental cytokines production from peripheral lymphocytes through a macrophage-mediated inhibition (Sotgiu et al., 2006). Two decades ago, Chaumeil et al. (1994) proposed colostrum to treat severe dryness and problematic eye lesions showing a beneficial effect. Our data showed that topical 2-fucosyl-lactose is an effective molecule when tested in experimental atropine-induced dry eye model in rabbit. In particular, fucosyl-lactose significantly inhibited atropine-induced tear volume (e.g., 1% fucosyl-lactose-treated group 10.75 vs. 3.75 mm vehicle-treated group) and TBUT (e.g., 1% fucosyl-lactose-treated group 34.5 vs. 4.75 s vehicle-treated group) reduction after 24 h in a dose-dependent manner. Because the mechanisms underlying the therapeutic effects of topical 2-fucosyl-lactose administration remain unclear, further research, such as controlled studies with placebo or with current treatment, is required to establish which is the added value of using this sugar in the treatment of dry eye disease. However, a clue on mechanism/s come from a recent study where the authors demonstrated that 2-fucosyl-lactose inhibits LPS-mediated inflammation in an *in vitro* model through a sophisticated signaling pathway (He et al., 2014). These authors showed that 2-fucosyl-lactose inhibits membrane-bound CD14 expression, while increasing soluble form of CD14 in the supernatant and the internalization of membrane form of CD14. Further, 2-fucosyl-lactose increases the amounts of negative regulatory molecules, including p-Akt, p-p38, suppressors of cytokine signaling 2 (SOCS2), phosphorylated signal transducer and activator of transcription 3 (pSTAT3) and IκB, while repressing pErk and NF-κB levels (He et al., 2014).

In conclusion, our findings support the hypothesis that 2-fucosyl-lactose, a natural sugar present in the human colostrum, may be useful in clinical practice to manage ocular surface diseases.

### Conflict of Interest Statement

The authors declare that the research was conducted in the absence of any commercial or financial relationships that could be construed as a potential conflict of interest.
